# Low Mother-to-Child CCL22 Chemokine Levels Are Inversely Related to Mite Sensitization and Asthma in Early Childhood

**DOI:** 10.1038/s41598-018-24523-8

**Published:** 2018-04-16

**Authors:** Chih-Yung Chiu, Kuan-Wen Su, Ming-Han Tsai, Man-Chin Hua, Sui-Ling Liao, Shen-Hao Lai, Li-Chen Chen, Tsung-Chieh Yao, Kuo-Wei Yeh, Jing-Long Huang

**Affiliations:** 1 Department of Pediatrics, Chang Gung Memorial Hospital at Keelung and Chang Gung University College of Medicine, Taoyuan, Taiwan; 2 Division of Pediatric Pulmonology, Chang Gung Memorial Hospital, College of Medicine, Chang Gung University, Taoyuan, Taiwan; 3 Community Medicine Research Centre, Chang Gung Memorial Hospital, Keelung, Taiwan; 4 Division of Allergy, Asthma, and Rheumatology, Department of Pediatrics, Chang Gung Memorial Hospital, and Chang Gung University College of Medicine, Taoyuan, Taiwan

## Abstract

Few studies have addressed the mother-to-child transmission of Th2 immunity and the impact on the development of atopic diseases in early childhood. We investigated 186 children who were followed-up regularly for 4 years in a birth cohort study. The levels of Th2 related chemokine (C-C motif) ligand 17 (CCL17) and CCL22 were quantified in cord blood and at 1.5 years-of-age using multiplex Luminex kits. The levels of 125 pairs of CCL17 and CCL22 chemokines from birth to 1.5 years were recorded in this study. Using *K*-means clustering, only the declining trend of CCL22 levels was separately clustered (cluster A, n = 51; cluster B, n = 46; cluster C, n = 28). Mothers of children with higher CCL22 chemokine levels at birth were significantly more likely to display *Dermatophagoides pteronyssinus* sensitization. A lower CCL22 level at birth with a slight rise during infancy was associated with higher prevalence of mite sensitization and a higher risk of asthma at 3 years-of-age (*P* = 0.014). In conclusion, low mother-to-child Th2-associated chemokine CCL22 levels appear to be inversely related to mite sensitization and the risk of asthma development in early childhood.

## Introduction

The imbalance in the T helper (Th)1/Th2 cell-related immune response plays a key role in the clinical expression of allergy and/or asthma^1^. In children, atopic diseases appear early in life, presumably as a result of skewed Th2-mediated immunity^2^. The neonatal immune system is Th2-skewed and most likely functions to divert the response of maternal immunity away from damaging Th1-mediated immunity toward fetal alloantigens^3^. This Th2-skewed immunity at birth even appears to be associated with infants later developing allergic diseases^4^. However, the changes of Th2-skewed immunity during infancy and its impact on atopic disease development later in life are unclear.

The aim of this study was to determine the levels of Th2-associated chemokines CCL17 and CCL22 at birth and 1.5 years-of-age in children from a birth cohort in the Prediction of Allergies in Taiwanese Children (PATCH) study. The changes of Th2-associated chemokine levels during infancy were clustered, and their relationships between allergic sensitization from mother to child and atopic disease development during early childhood were assessed.

## Results

### Study Population

A total of 258 children were recruited initially. Of these, 186 (72.1%) children who completed a 4-year follow-up at the clinic were enrolled in this study. CCL17 and CCL22 chemokines were measured in 125 children with data of cord blood and serum samples at 1.5 years-of-age. There were no significant differences in the baseline characteristics among these 125 children with paired samples, 186 children, and all 258 children^5^. Furthermore, no significant difference in the prevalence of atopic diseases was found between children with and without blood samples during the follow-up period^6^. At 4 years-of-age, atopic diseases including eczema, rhinitis, and asthma were physician-diagnosed in 14, 53, and 19 of the 125 children respectively.

### Clustering Analysis of CCL17 and CCL22 Levels during Infancy

**Figure 1:**
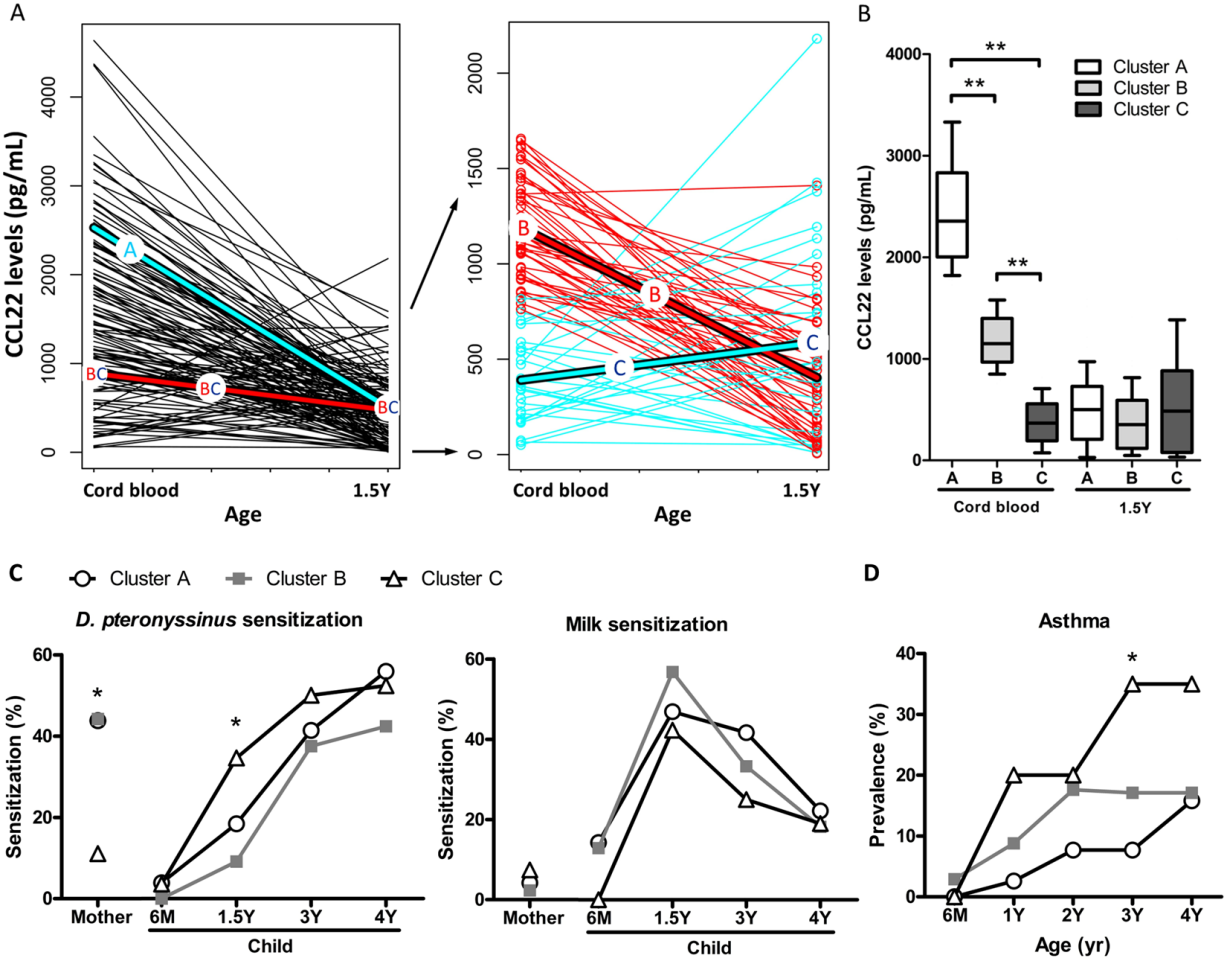
Clustering of CCL22 levels during infancy and their relationships between allergic sensitization and atopic diseases at different years of age. The pattern of CCL22 levels from birth to age 1.5 clustering by using *K*-means method in R software (**A**). Comparisons and differences between CCL22 clustering (**B**) and their associations between allergen sensitization to mite and milk (**C**) and asthma (**D**) at different years of age. The box-plot showing the median and the 10th, 25th, 75th and 90th percentile. Bonferroni-adjusted *P*-values referred to the comparisons of *D. pteronyssinus* sensitization at age 1.5 [cluster C vs. cluster B] and allergic asthma at age 3 [cluster C vs. cluster A] are indicated by the marker. *P < 0.05; **P < 0.01.

*K*-means clustering was performed in the 125 paired samples concerning CCL17 and CCL22 chemokine levels from birth to 1.5 years-of-age using R software. Both CCL17 and CCL22 levels were the highest at birth and then decreased. The decline of CCL17 levels was not clustered into groups in any specific pattern. However, the CCL22 level change was significantly stratified into three clusters (Fig. 1A). Cluster A (n = 51) showed a sharp decline in CCL22 levels from a median of 2,356.5 pg/mL to 500.6 pg/mL. Cluster B (n = 46) showed a moderate decline in CCL22 levels from a median of 1,149.6 pg/mL to 351.8 pg/mL. Cluster C (n = 28) showed a slight rise in CCL22 levels from a low median of 366.4 pg/mL to 486.3 pg/mL (Fig. 1B). Table 1 presents the baseline characteristics of the 125 children with paired samples in relation to CCL22 clustering. Mothers with a lower prevalence of mite sensitization was significantly associated with children with lower serum CCL22 status in cluster C (*P* = 0.008), but no other significant difference was found in the three clusters.

**Table 1:** Baseline characteristics of 125 children in relation to CCL22 clustering during infancy.

	Cluster A (n = 51)	Cluster B (n = 46)	Cluster C (n = 28)	*P*-value
**Family**				
Maternal atopy	24 (47.1%)	19 (41.3%)	10 (35.7%)	0.610
Food sensitization	2 (4.2%)	1 (2.3%)	2 (7.4%)	0.590
Mite sensitization	21 (43.8%)	19 (44.2%)	3 (11.1%)	0.008
Paternal atopy	29 (56.9%)	24 (52.2%)	18 (64.3)	0.594
Parental smoking	32 (62.7%)	24 (52.2%)	11 (39.3%)	0.131
Older siblings	17 (33.3%)	20 (43.5%)	15 (53.6%)	0.207
Household income				0.299
Low, ≤ 500,000 NTD	18 (35.3%)	22 (43.1%)	11 (21.6%)	
Medium, 500,000–1,000,000 NTD	20 (43.5%)	22 (47.8%)	4 (8.7%)	
High, > 1,000,000 NTD	11 (39.3%)	15 (53.6%)	2 (7.1%)	
**Infant**				
Sex				0.920
Male	32 (62.7%)	27 (58.7%)	17 (60.7%)	
Female	19 (37.3%)	19 (41.3%)	11 (39.3%)	
Maternal age (yr)	31.0 ± 4.3	30.4 ± 4.2	30.9 ± 3.9	0.735
Gestational age (wk)	38.7 ± 1.3	37.9 ± 2.0	37.9 ± 1.7	0.058
Birth BMI (kg/m^2^)	12.3 ± 1.1	12.3 ± 1.4	13.7 ± 3.5	0.342
Breastfeeding				0.844
Exclusive	23 (45.1%)	18 (39.1%)	9 (32.1%)	
Partial	16 (31.4%)	15 (32.6%)	10 (35.7%)	
Formula	12 (23.5%)	13 (28.3%)	9 (32.1%)	

Data shown are mean ± S.D. or number (%) of patients as appropriate. NTD, new Taiwan dollar; yr, year; wk, week; BMI, body mass index.

### Association between CCL22 Clustering and Allergic Sensitization

Compared with cluster C, CCL22 chemokine levels at birth in cluster A and B were significantly higher in children born to mothers with a higher prevalence of *D. pteronyssinus* sensitization. Comparisons and differences between the three clusters concerning CCL22 chemokine levels and allergen sensitization at different ages are summarized in Fig. 1C. A significantly higher prevalence of *D. pteronyssinus* sensitization was found in children in cluster C compared with children in cluster B at 1.5 years-of-age. There were no significant differences in the association between CCL22 clusters and total serum IgE levels or allergen sensitization to egg white, milk, and wheat.

### Association between CCL22 Clustering and Atopic Diseases

CCL22 clustering during infancy was not associated with the risk of eczema and allergic rhinitis throughout early childhood. However, compared with children in cluster A, a significantly higher prevalence of asthma was found in children in cluster C at the age of 3 years (Fig. 1D). Multiple logistic regression analysis revealed that children with a slight rise in CCL22 levels in cluster C were more likely to suffer from asthma at 3 years-of-age (odds ratio, 6.46; 95% confidence interval, 1.45–28.78; *P* = 0.014).

## Discussion

Atopic diseases are characterized by a T helper (Th) 2-skewed immunity. However, little is known concerning the transmission of Th2 immunity from mother to child and its impact on the development of atopic disease. This study has demonstrated that children with Th2-skewed immunity at birth are significantly more likely to have been born to mothers with mite sensitization but are not associated with the risk of atopic disease development later in life. However, an inverse association between cord blood CCL22 levels and allergic sensitization risk in asthma suggests that the antibodies to allergens of maternal influences may induce tolerance and protection from allergic asthma in early childhood.

The development of atopy is favored by a shift towards Th2 immunity that features the production of IgE antibodies^2^. Circulating chemokine levels in infants are evidently higher than cytokine levels and have reportedly been used as biomarkers for atopic diseases^7^. Th2-associated CC chemokine ligand (CCL) including CCL17 and CCL22 activate the chemokine receptor CC chemokine receptor 4 (CCR4) which plays a key role in allergen-driven Th2 cell accumulation in asthmatic airways^8^. CCL22 is a dominant ligand of CCR4 over CCL17, which may explain the present and previous observations relating CCL22 levels rather than CCL17 levels, at birth with the risk for developing atopic disease later in life^9,10^.

The maternal environment during pregnancy promotes an initial Th2-skewed immune response in the offspring. Failure of Th2-silencing after birth may underlie the development of Th2-mediated atopic diseases^11,12^. In this study, the mothers of infants born with higher CCL22 levels (>1000 pg/mL) were more likely to display mite sensitization, supporting the view that the mother plays a crucial role in mediating the development of immunity prior to birth and during infancy. After birth, CCL22 levels declined sharply without showing differences in levels at 1.5 years-of-age, indicating that the influence of an allergic mother may not be enough to delay the normal transition to a non-allergic immune response during infancy.

Circulating cord blood CCL22 levels have been associated with IgE production and allergic sensitization in early childhood^13^. However, the influence of Th2-skewed immunity at birth on atopy appears to be apparent before 2 years of age, but not thereafter^10^. In this study, a lower CCL22 level at birth (<500 pg/mL) with a slight rise during infancy was related to higher prevalence of mite sensitization and asthma later in life. These findings suggest that a skewed Th2 neonatal immunity may not be sufficient to increase the risk of the development of atopic diseases. In contrast, constant exposure to environmental stimuli during growth may reinforce or antagonize the maternal influence and play an important role in allergic immune response to inhaled allergens.

A number of factors including prenatal and postnatal exposure to allergic triggers have been identified to increase the susceptibility to asthma^14–16^. However, maternal allergies are poor predictors of childhood atopic asthma^17^. Conversely, in the present study, children born with lower Th2 responses appeared to be at higher risk for childhood asthma. The transmission of asthma risk from mother to child likely involves prenatal factors transmitted across the placenta^12^. Protective benefits of early allergen exposure in asthma have been replicated in multiple birth cohorts and were recently proven in food allergy prevention trials^18,19^. The inverse association between Th2-skewed immunity at birth and atopic asthma in early life observed in this study may be explained by the maternal transfer of an antigen inducing tolerance and protection from allergic asthma^20^.

The major limitation of this study is the small sample size of the birth cohort, which limited the statistical power. Furthermore, Th2-associated chemokines may not totally represent the biological activities of Th2 cytokines which play an important role in the pathophysiology of allergic diseases, including asthma. The strengths of this study are its longitudinal analysis and follow-up at very close intervals, allowing a systematic observation of the sensitization to allergen and development of atopic diseases.

In conclusions, our study has demonstrated that high levels of Th2-associated chemokine CCL22 at birth appear to be from mite-sensitized mothers and decline sharply during infancy. This influence may not delay the normal transition of immunity and may not be associated with the risk of development of atopic disease in the offspring. In contrast, children born with lower CCL22 levels appear to be inversely associated with the risk of allergic asthma later in life. These findings suggest the maternal transfer of antibodies to allergens may play a role in inducing tolerance and protection from allergic asthma. A larger study will be required to investigate these associations more comprehensively.

## Methods

### Patients and Data Collection

Children recruited in a birth cohort study launched at Taiwan and completed a 4-year follow-up were enrolled. Detailed descriptions of the subject recruitment of this birth cohort study were reported previously^14,21^. Detailed information on potential confounding variables for atopic diseases including child’s age, sex, family atopy history, passive smoking, household income, and history of breastfeeding were collected and analyzed. This study was approved by the Ethics Committee of Chang Gung Memorial Hospital (No. 103-6236A3). All experiments in this study were performed in accordance with the relevant guidelines and regulations and written informed consent was obtained from the parents or guardians of all study subjects.

### Evaluation and Diagnosis of Atopic Diseases

The phenotypes of atopic diseases were physician-diagnosed and evaluated by the same pediatric pulmonologist at clinics. The diagnosis of atopic diseases including eczema, allergic rhinitis and asthma was described as in our previous study^5,6,10^. Eczema was defined as a pruritic rash over the face and/or extremities with a chronic relapsing course; allergic rhinitis was defined as having a history of sneezing, nasal congestion, itching, and rhinorrhea, or current use of medication for these symptoms; asthma was diagnosed based on the Global Initiative for Asthma guidelines with the presence of a recurrent wheeze, or current use of asthma medication^22–24^.

### Measurement of Th2-Associated Chemokine Levels and Clustering Analysis

The levels of CCL17 and CCL22 chemokines were quantified in cord blood and serum at age 1.5 by multiplex Luminex kits (Bio-Rad Laboratories, Hercules, CA), according to the manufacturer’s instructions as our previously research described^10^. All samples were analyzed in duplicates with the coefficient of variation (CV) < 10% with lower limit of detection of 1.7 pg/mL for CCL17 and 0.9 pg/mL for CCL22 respectively. For clustering, CCL17 and CCL22 chemokine levels at birth and age 1.5 were imported into R software (Version 3.3.1). *K*-means clustering was then used to group CCL17 and CCL22 levels into discrete and stable clusters of time series data from birth to age 1.5 using R software.

### Total serum and Allergen-Specific Immunoglobulin E

As described in our previous study^5^, allergen-specific IgE was determined using a commercial assay for IgE (ImmunoCAP Phadiatop Infant; Phadia) for a mixed of three most common food allergens (egg white, milk and wheat) and inhalant allergens (*D. pteronyssinus*, *D. farina* and *C. herbarum*) in Taiwan^25,26^. Allergen sensitization was defined as values ≥ 0.35 kU/L^27^.

### Covariates

Confounders associated with atopic disease development were included and adjusted in the multiple logistic regression analysis. Factors including child’s sex, gestational age and maternal age at delivery, prenatal passive smoke exposure, maternal atopy, older siblings at birth, breastfeeding patterns, and household income were collected and analyzed.

### Statistical analysis

*K*-means clustering of CCL17 and CCL22 chemokine levels was performed using R software. Comparisons of baseline characteristics and allergic sensitization categorized by CCL17 and CCL22 clusters were performed with univariable parametric and nonparametric tests such as ANOVA, χ^2^, Fisher’s exact test, and Kruskal–Wallis rank sum test. The Bonferroni post hoc test was used for pairwise comparisons among the rank means of three or more groups. Multiple logistic regression analysis was used to determine the associations of CCL17 and CCL22 clusters and atopic diseases by adjusting the confounding factors. Statistical analysis was performed using the Statistical Package for the Social Sciences (SPSS Statistics for Windows Version 22.0; Armonk, NY, USA), and GraphPad Prism software (GraphPad Software Inc. Version 5.01; San Diego, CA, USA) was used to graph data. All statistical hypothesis tests were two-tailed, and a *P*-value of less than 0.05 was considered significant.
